# A novel approach to Verify air gap and SSD for proton radiotherapy using surface imaging

**DOI:** 10.1186/s13014-019-1436-4

**Published:** 2019-12-11

**Authors:** Xiao Wang, Chi Ma, Rihan Davis, Rahul R. Parikh, Salma K. Jabbour, Bruce G. Haffty, Ning J. Yue, Ke Nie, Yin Zhang

**Affiliations:** Department of Radiation Oncology, Rutgers Cancer Institute of New Jersey, Rutgers Robert Wood Johnson Medical School, Rutgers, The State University of New Jersey, New Brunswick, NJ USA

**Keywords:** Proton therapy, Air gap, SSD, Surface imaging

## Abstract

**Purpose:**

To develop a novel approach to accurately verify patient set up in proton radiotherapy, especially for the verification of the nozzle – body surface air gap and source-to-skin distance (SSD), the consistency and accuracy of which is extremely important in proton treatment.

**Methods:**

Patient body surfaces can be captured and monitored with the optical surface imaging system during radiation treatment for improved intrafraction accuracy. An in-house software package was developed to reconstruct the patient body surface in the treatment position from the optical surface imaging reference capture and to calculate the corresponding nozzle – body surface air gap and SSD. To validate this method, a mannequin was scanned on a CT simulator and proton plans were generated for a Mevion S250 Proton machine with 20 gantry/couch angle combinations, as well as two different snout sizes, in the Varian Eclipse Treatment Planning Systems (TPS). The surface generated in the TPS from the CT scan was imported into the optical imaging system as an RT Structure for the purpose of validating and establishing a benchmark for ground truth comparison. The optical imaging surface reference capture was acquired at the treatment setup position after orthogonal kV imaging to confirm the positioning. The air gaps and SSDs calculated with the developed method from the surface captured at the treatment setup position (VRT surface) and the CT based surface imported from the TPS were compared to those calculated in TPS. The same approach was also applied to 14 clinical treatment fields for 10 patients to further validate the methodology.

**Results:**

The air gaps and SSDs calculated from our program agreed well with the corresponding values derived from the TPS. For the phantom results, using the CT surface, the absolute differences in the air gap were 0.45 mm ± 0.33 mm for the small snout, and 0.51 mm ± 0.49 mm for the large snout, and the absolute differences in SSD were 0.68 mm ± 0.42 mm regardless of snout size. Using the VRT surface, the absolute differences in air gap were 1.17 mm ± 1.17 mm and 2.1 mm ± 3.09 mm for the small and large snouts, respectively, and the absolute differences in SSD were 0.81 mm ± 0.45 mm. Similarly, for patient data, using the CT surface, the absolute differences in air gap were 0.42 mm ± 0.49 mm, and the absolute differences in SSD were 1.92 mm ± 1.4 mm. Using the VRT surface, the absolute differences in the air gap were 2.35 mm ± 2.3 mm, and the absolute differences in SSD were 2.7 mm ± 2.17 mm.

**Conclusion:**

These results showed the feasibility and robustness of using an optical surface imaging approach to conveniently determine the air gap and SSD in proton treatment, providing an accurate and efficient way to confirm the target depth at treatment.

## Background

Wilson theorized that fast protons could be used for treating deep-seated tumors while sparing adjacent normal tissues due to the unique physical properties in 1946 [1], and according to the PTCOG website (https://www.ptcog.ch) as of December 2017, more than 170,000 patients worldwide have been treated with proton therapy. The advantage of proton radiotherapy is that along proton beam path, high dose could be tailored to cover the target, while spilling very little dose to normal tissues beyond the target [2–4]. This treatment requires high precision and accuracy in treatment delivery, not only in beam quality itself but also in the radiological equivalent depth of the target along the beam path direction. While the range of proton beam is set in treatment planning system (TPS), any change in the radiological equivalent depth during treatment could potentially cause under-coverage of the target, or shifting of high doses to the normal tissues.

Currently, volumetric imaging, using cone-beam computed tomography (CBCT) or CT-on-rails, and 2D orthogonal kilovoltage (kV) imaging are the available options for image guidance in proton therapy to ensure patient position before treatment [5–7]. Although these IGRT tools are very useful in positioning the target volume correctly relative to the treatment machine, it is critically important to acquire the target depth information to ensure correct coverage. However, for proton centers where orthogonal kV imaging is the only image guidance option, this target depth information cannot be directly and conveniently provided. The alignment of target to machine isocenter can be achieved with orthogonal kV imaging, but target depth change, for example, weight loss, cannot be detected with just orthogonal kV imaging. On the other hand, the physical depth change will be reflected in source-to-skin distance (SSD) change after alignment of target to machine isocenter, keeping source-axis distance (SAD) the same. Therefore, the target depth change can be detected through the change in SSD, as well as the air gap, defined as the distance between the end of compensator and the body surface of patients closest to the end of the compensator inside the treatment area. Since many of the existing proton therapy systems are not yet equipped with the SSD indicator, checking the air gap consistency and accuracy is more convenient, and is critical in proton therapy. At many proton centers, the current approach to verify the air gap at the time of treatment is to manually measure the closest distance from the end of compensator to patient surface inside the snout area with a ruler, which can be subjective and inefficient.

The optical surface imaging systems have become widely available to radiation oncology departments and have been routinely used for the purpose of patient setup and motion management. With this type of system, the patient body surface position can be acquired and/or monitored during set up and treatment for improved accuracy [8]. It is conceived that the acquired surface coordinates can be directly used to compute and derive the air gap for proton patient treatment without resorting to any manual measurement tools, and that the SSD can also be obtained in the same process. The purpose of this work is to develop an optical surface imaging based novel approach to accurately and automatically derive values of the air gap and SSD at patient setup and treatment in proton therapy.

## Methods

A Mevion S250 (Mevion Medical Systems, Littleton, MA, USA) proton system has been clinically used to treat patients since 2015 in our department. The proton system is a single-room, gantry-mounted passive scattering proton therapy machine, equipped with a 6D robotic couch and a pair of orthogonal planar kV X-ray imagers for image guided patient setup [9]. It includes a synchrocyclotron mounted on a gantry that rotates from − 5° to 185° around the isocenter. An optical surface imaging system AlignRT (VisionRT, London, United Kingdom) was installed in our proton center in 2017 and has been routinely used to guide treatment setup and treatment. It is a video-based three-dimensional (3D) surface imaging system, and is used to image the skin surface of a patient in 3D before and during radiotherapy treatment. The system utilizes a combination of light projectors and high-definition optical cameras to generate a 3D map of a patient’s topography. Projectors and optical cameras are mounted in pods on the ceiling of the treatment room and a typical installation has 3 pods to allow visualization of the patient in different couch positions or gantry angles. The system is calibrated relative to the isocenter of the proton machine, so the geometric coordinates of the generated 3D map of patient body surface can be readily obtained relative to the machine isocenter.

**Fig. 1:**
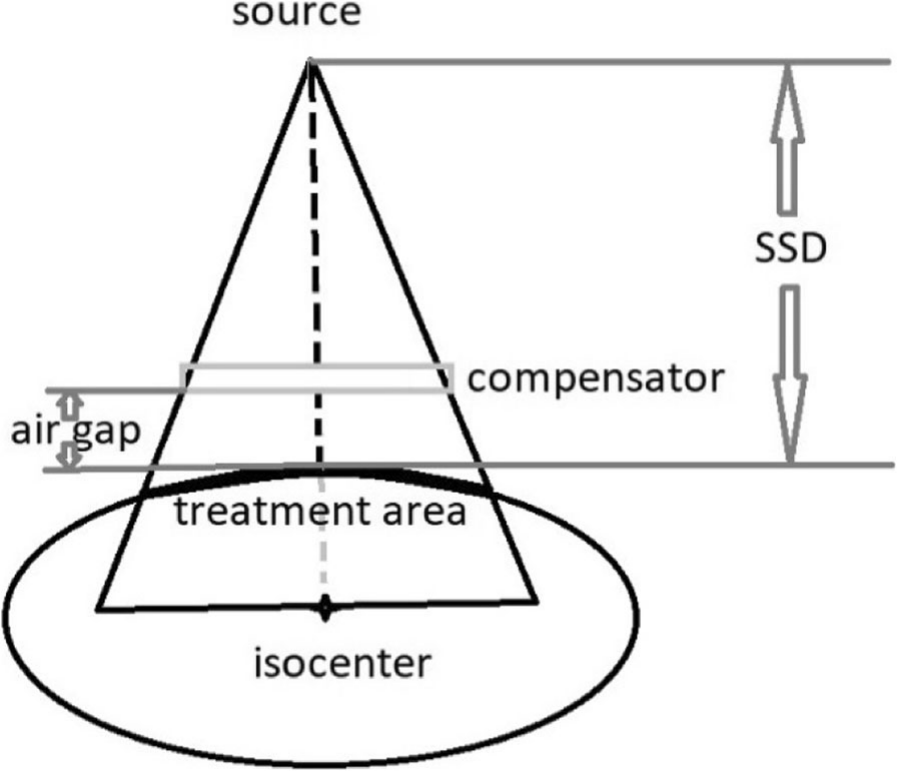
Diagram of air gap and SSD definition. The treatment area is highlighted in bold line

**Fig. 2:**
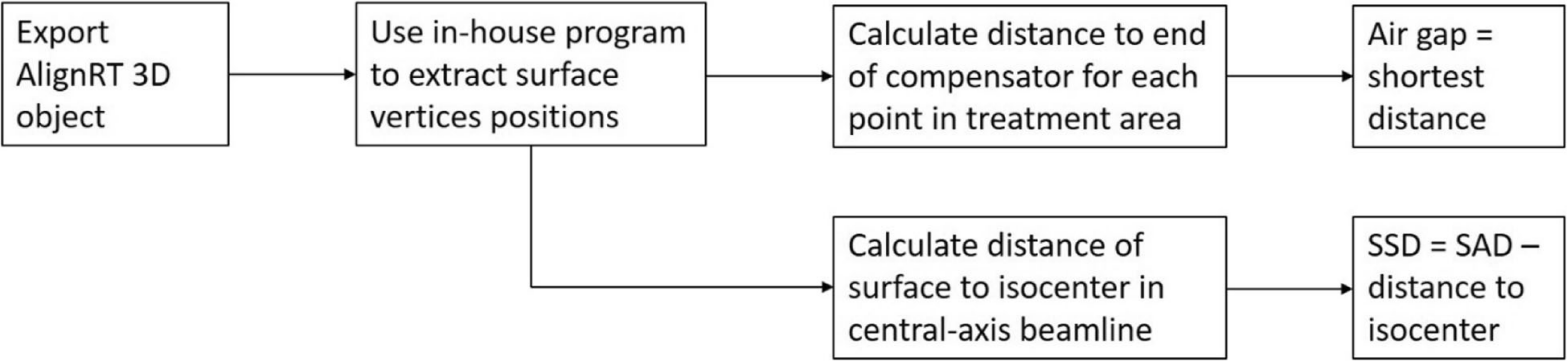
Workflow of our in-house software package

An in-house Matlab program was developed to reconstruct surface from the surface information captured by AlignRT. First, this program renders the 3D surface by extracting vertices of a triangular mesh from AlignRT 3D model file (.obj). The position information of these vertices are then used to calculate the distance between the end of a compensator and all the surface points in the treatment area. The shortest distance from patient surface to the end of compensator inside the treatment field would be the air gap, as shown in Fig. 1. The geometric coordinates of the machine relative to the isocenter, including the virtual source, snout and compensator, was imported to the in-house software package in DICOM format. The treatment area was determined inside a pyramid. The top of the pyramid is the source. The bottom of the pyramid is a rectangle on the isocenter plane, perpendicular to the beam path direction. The size of the rectangle is the size of the inner perimeter of the snout. Therefore, only points that fall in the pyramid were accounted for in the calculation. The calculations traced central-axis beamline from the nominal source of the proton machine to the isocenter. After obtaining the distance from the isocenter to patient surface along the central-axis beamline, the SSD could be derived by subtracting this distance from the nominal SAD. The nominal SAD for the Mevion S250 machine is 200 cm. With the surface geometric coordinate information acquired by AlignRT software, the air gap and SSD of each proton beam were then automatically computed and derived. The workflow of the in-house Matlab program is outlined in Fig. 2.

**Fig. 3:**
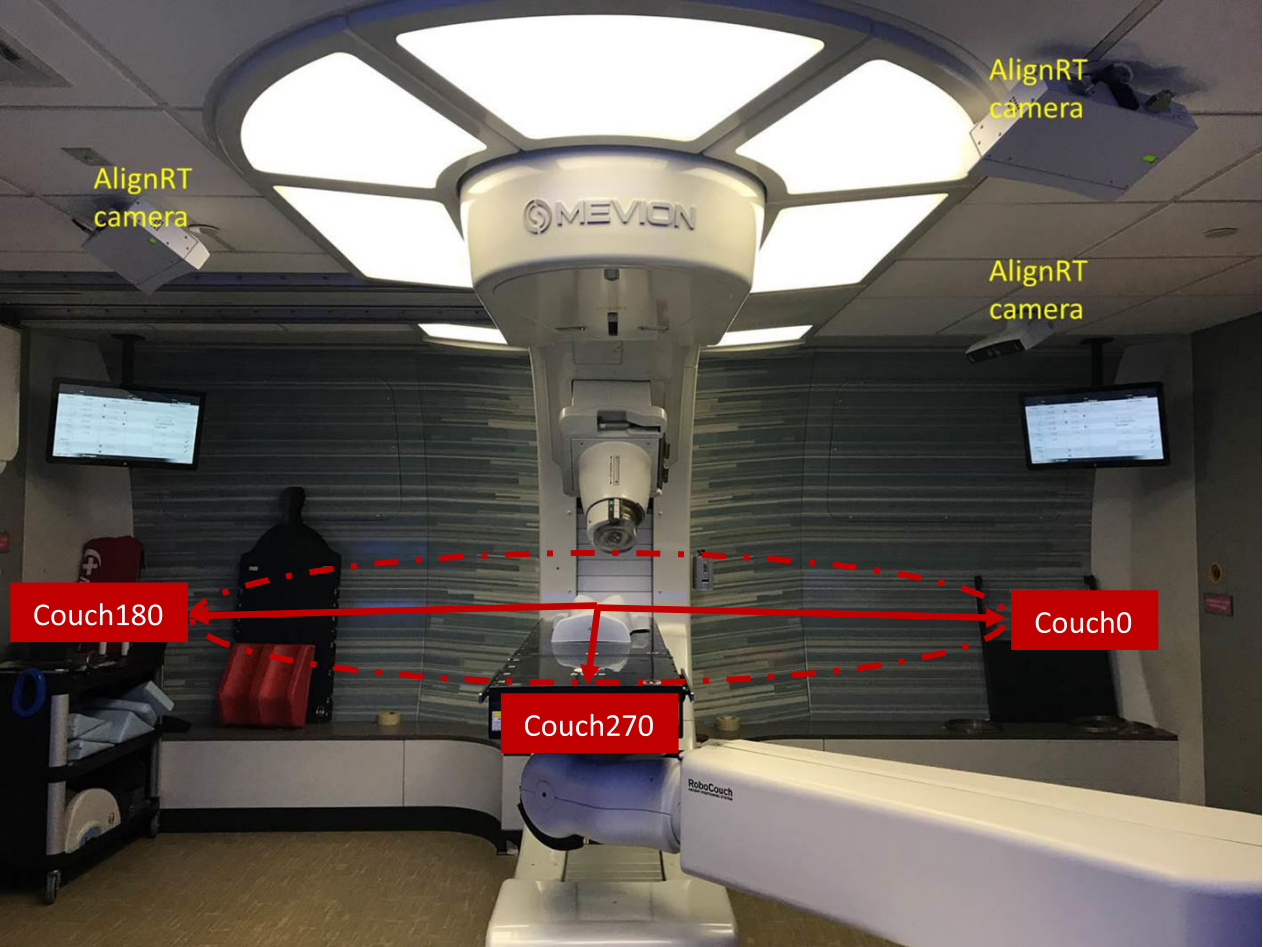
Room set up of the Mevion S250™ proton therapy machine equipped with AlignRT. The couch angles are labeled in the figure. The couch angle for the mannequin set up shown in the figure is 270 degrees

In order to validate this method, CT images of a mannequin (as shown in Fig. 3) was acquired on a GE LightSpeed 16 CT scanner (GE Medical System, Milwaukee, WI, USA). Several proton plans were generated on the Mevion S250 Proton machine with various gantry and couch angle combinations, as well as different snout sizes (14 cm for the small snout, and 25 cm for the large snout), in Varian Eclipse V11.0 TPS (Varian Medical Systems, Palo Alto, CA, USA). The gantry angles used in the plans were 0, 30, 45, 60 degrees, and couch angles were 180, 225, 270, 315, 0 degrees. The body contour generated from CT scanning was imported into AlignRT system for phantom set up and for the benchmark validation of the developed method. When setting up the mannequin in the treatment room, after positioning verification using the orthogonal kV imaging, an AlignRT reference capture was taken at the set up position, which recorded the surface geometric coordinate information of the mannequin. The mannequin set up is shown in Fig. 3.

**Fig. 4:**
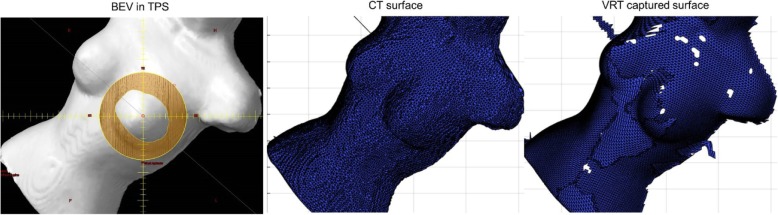
An example of comparison of BEV in TPS, reconstructed CT surface, and AlignRT (VRT) captured surface

The captured surface at the setup position (VRT surface), along with the CT body surface (CT surface) in the AlignRT system, was exported from the AlignRT system and imported into the in-house software package. The CT body surface that was imported to the in-house software package from the AlignRT system was also used to compute the air gap and SSD for the purpose of validating and establishing the benchmark accuracy of the developed methodology. Theoretically and ideally, the computed air gap and SSD derived from the imported CT body surface, compared to the corresponding air gap and SSD in the treatment planning system, should be exactly matched. However, due to digital round-off, they could slightly differ from each other. An example of comparison of beam’s eye view (BEV) in TPS, the CT body surface and VRT body surface is shown in Fig. 4. The values of air gap and SSD at each treatment angle were calculated from the CT surface, and compared against TPS values to establish the benchmark accuracy of the digital round-off. The values of the air gap and SSD calculated from VRT surface were computed and compared to those calculated in TPS to establish the benchmark accuracy of the developed methodology.

The developed methodology was also retrospectively evaluated using clinical data. In our clinic, the AlignRT system is routinely used for patient setup and motion monitoring at proton treatment. The VRT body surfaces are routinely acquired for the patient treatments to which the AlignRT system is applied. The evaluation was conducted for 10 clinical proton patients. Among the 10 patients, 5 were liver patients, 2 were prostate patients, 2 were mediastinum patients, and 1 was a breast patient. In the clinical practice involving the AlignRT system in our clinic, before treatment, one set of patient body surface is always captured after the patient position (relative to the isocenter) is corrected and verified with the orthogonal kV image pairs. The acquired body surfaces for the 10 clinical cases were used to derive the values of the air gap and SSD for each of treatment fields using our method, and were compared to corresponding TPS values, using the same approach as above with the mannequin phantom. The values of the air gap and SSD were only calculated for the treatment fields with anterior angles since patient posterior surface could not be captured by the AlignRT system due to the camera arrangement.

## Results

**Fig. 5:**
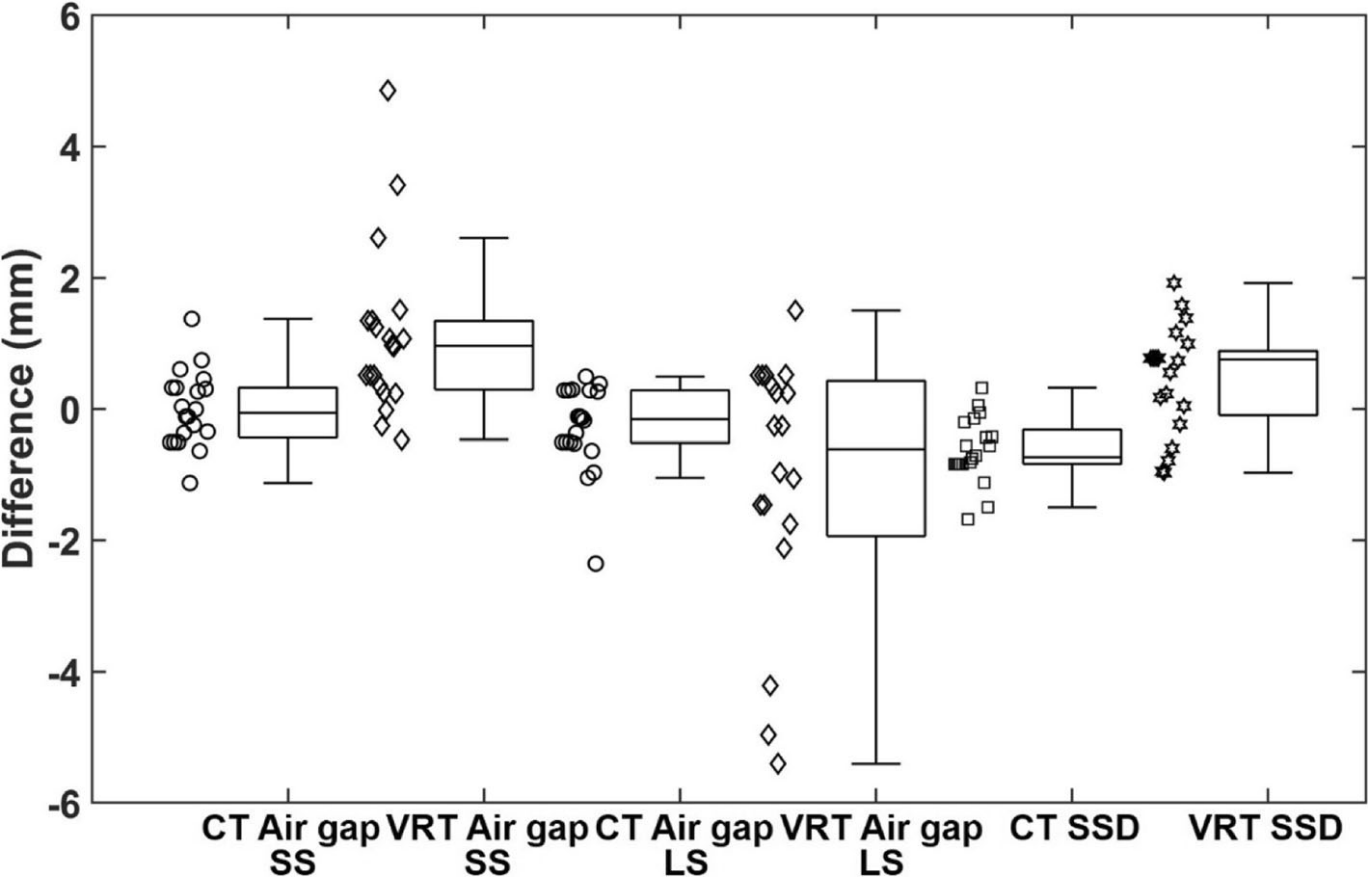
Box and whisker plot of differences between calculated and planned air gap and SSD for CT surface and VRT captured surface for phantom with small snout (SS) and large snout (LS) (*n* = 20)

The result comparison of the air gap and SSD for the phantom is presented as a box and whisker plot in Fig. 5. Among all 20 fields with different gantry and couch angle combinations, using the CT body surface, the absolute differences in the air gap were 0.45 mm ± 0.33 mm for small snout, and 0.51 mm ± 0.49 mm for large snout, and the absolute differences in SSD were 0.68 mm ± 0.42 mm regardless of snout size. Using the VRT body surface, the absolute differences in the air gap were 1.17 mm ± 1.17 mm and 2.1 mm ± 3.09 mm for the small and large snouts, respectively, and the absolute differences in SSD were 0.81 mm ± 0.45 mm. The large discrepancy happened where there was sharp surface gradient at the snout boundary.

**Fig. 6:**
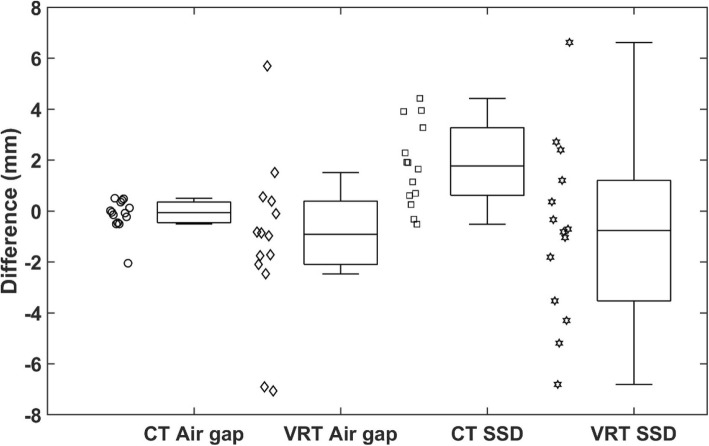
Box and whisker plot of differences between calculated and planned air gap and SSD for CT surface and VRT captured surface for patients (*n* = 14)

Figure 6 shows the comparison of the air gap and SSD for the 10 clinical patients in a box and whisker plot. The patient data is presented without differentiation of snout size since the previous phantom result (Fig. 5) did not show significant differences between the small and large snouts. Among all 14 treatment fields, using the CT body surface, the absolute differences in the air gap were 0.42 mm ± 0.49 mm, and the absolute differences in SSD were 1.92 mm ± 1.4 mm. Using the VRT body surface, the absolute differences in the air gap were 2.35 mm ± 2.3 mm, and the absolute differences in SSD were 2.7 mm ± 2.17 mm.

## Discussion

The values of the air gap and SSD calculated from the CT body surfaces with the developed methodology and calculation program agreed well with the corresponding TPS values within about 0.5 mm, validating the methodology with the digital round-off accuracy of about 0.5 mm. The values of the air gap and SSD calculated from the phantom VRT surfaces with our program are also in agreement with the TPS values within about 2 mm, validating the feasibility of utilizing this program to estimate the air gap and SSD with an accuracy of about 2 mm. The deviations calculated from the VRT body surface of the phantom were larger than those calculated from the CT body surface of the phantom, mainly caused by the limitation of the camera imaging accuracy compared to CT images. For the clinical patients, the result deviations derived from the VRT body surfaces were larger, which reflects the fact that the target depths may have exhibited some changes at treatment. The accuracy of the target depth calculations from the air gaps and SSDs can be dependent on multiple factors, including the patient setup accuracy, breathing motion, patient anatomical change, among others. In Fig. 6, there are a few data points showing ~ 6–7 mm air gap differences, indicating that there could be anatomical changes during the surface imaging capture with other possible causes, which were likely caused by factors such as sharp surface gradient at the snout boundary and breathing motion, etc. On the other hand, this proposed method is more sensitive than simple weight loss measure since it can detect the target depth changes caused not only by weight loss or gain but also the target and organ movement and other factors. The detected difference can be used as an indicator for additional evaluation actions and measures such as re-CT scan or adaptive re-plan.

The nature of how proton beam deposits energy along its beam path makes it essential to keep the radiological equivalent path length of proton beam consistent through a course of treatment. Otherwise, significant dose deviation may take place, leading to underdosing the target volume or delivering unplanned dose to organs at risk. However, the conventional 2D orthogonal kV imaging IGRT technology may help on the alignment of the target but is often inadequate to detect the target depth change along the proton beam path. Although 3D volumetric IGRT technologies, such as CBCT, may serve for the both purposes, the availability of the volumetric IGRT technologies to the existing proton centers is still limited. Recently, our group has published our experience using automatic measurements of proton air gap based on orthogonal kV imaging with radiopaque wires [10]. The current study is a further step and additional approach utilizing the optical surface monitoring device, which is already in clinical use for proton treatment and provides full surface information, to automatically derive the values of the air gap and SSD without resorting to some surface surrogate such as radiopaque wires. By using the surface information acquired by the AlignRT system, the distances between the surface and the end of compensator, as well as SSD, can be calculated and verified against the values planned in the TPS. Instead of measuring only one point on the patient’s surface, the distances between the compensator and all the surface points in the treatment area can be measured and verified with the surface information acquired by the AlignRT system. This is a thorough validation of patient treatment set up and will be a sensitive approach to flag anatomy changes during proton therapy treatment.

The optical surface imaging-based approach for the derivation and verification of the air gap and SSD has limitations in the setting of radiation treatment using posterior fields due to the configuration of the AlignRT camera systems. The three AlignRT cameras are normally mounted on the ceiling in the treatment room and can only capture the patient’s surface from above. The surface that cannot be captured by the cameras would not be able to be used for air gap or SSD calculation. Additionally, based on the captured surface image, one can visually determine if the surface of interest for a posterior beam is blocked by the tabletop or not and whether it is suitable for the calculations of the air gap and SSD. Therefore, the air gap and SSD calculation for some posterior beams may not be feasible with this method if the surface area cannot be captured by the cameras. In the current study, posterior fields were excluded, and only anterior fields were presented for both the phantom and patient studies. At our center, at least 70% of the treatment fields come in anteriorly, as the posterior fields would go through couch and possibly couch rails. Additionally, with the proton machine in our clinic, the patient image guided setup couch position is not necessarily the treatment couch position. The patient setup is normally conducted with the couch at 270-degree position. After the patient positioning is confirmed by the 2D orthogonal imaging system, the couch is returned to the treatment position (the imaging coordinate system and the treatment coordinate system are automatically inter-related and associated by the proton system). The patient body surfaces were also captured by the AlignRT system at the setup position (couch at 270 degrees). Ideally, the surfaces should be captured at the treatment couch position.

Although the methodology was developed using the AlignRT system, the principle of the methodology can be applied to any other optical surface imaging system used in radiation oncology clinic. Additionally, the principle can also be applied to the radiation treatments using the conventional photon or electron beams for the clinics where volumetric IGRT technologies are not available. This proposed method would be of value for centers where their proton machines are not equipped with CBCT or treatment CT capability and it provides a sensitive measure to trigger additional evaluations and measures to ensure proper treatment and quality. However, the clinical tolerances for different action levels are site dependent as well as dependent on the planning range uncertainty margins and PTV margins, which may vary from institution to institution. Future studies on the tolerance levels are needed to explore the optimal action and measure when the deviations are detected.

## Conclusions

We have developed an optical surface imaging based methodology to automatically derive the values of the air gap and SSD for proton treatments. The methodology was evaluated and validated using both phantom and clinical patient data. The results show that it is feasible and robust to use the optical surface imaging approach to conveniently determine the air gap and SSD in proton treatment, with acceptable accuracy. This approach provides an objective and efficient way to confirm the physical target depth at treatment, in order to ensure desired target coverage and normal tissue sparing.

## Data Availability

The datasets used and analyzed during the current study are available from the corresponding author on reasonable request.
